# Assessment of the effectiveness of a small quantity lipid-based nutrient supplement on reducing anaemia and stunting in refugee populations in the Horn of Africa: Secondary data analysis

**DOI:** 10.1371/journal.pone.0177556

**Published:** 2017-06-07

**Authors:** Sarah Style, Melody Tondeur, Carlos Grijalva-Eternod, Josephine Pringle, Ismail Kassim, Caroline Wilkinson, Allison Oman, Carmel Dolan, Paul Spiegel, Andrew Seal

**Affiliations:** 1 Institute for Global Health, University College London, London, United Kingdom; 2 United Nations High Commissioner for Refugees, Geneva, Switzerland; 3 Emergency Nutrition Network, Oxford, United Kingdom; TNO, NETHERLANDS

## Abstract

Stunting and micronutrient malnutrition are persistent public health problems in refugee populations. UNHCR and its partner organisations implement blanket supplementary feeding programmes using a range of special nutritional products as one approach to address these issues. The evidence base for the efficacy and effectiveness of a small quantity lipid-based nutrient supplement, Nutributter^®^, in reducing stunting and anaemia is limited. Secondary data analysis was used to assess the effectiveness of Nutributter^®^ distribution on anaemia and stunting in children aged 6–23 months (programme target group) and 6–59 months (the standard age group sampled in routine nutrition surveys). Analysis was conducted using routine pre and post-intervention cross-sectional nutrition survey data collected between 2008–2011 in five refugee camps in Kenya and Djibouti. Changes in total anaemia (Haemoglobin<110g/L), anaemia categories (mild, moderate and severe), and stunting (height-for-age z-score <-2) were explored using available data on the Nutributter^®^ programme and contextual factors. A significant reduction in the prevalence of anaemia in children aged 6–23 months and 6–59 months was seen in four of five, and in all five camps, respectively (p<0.05). Reductions ranged from 12.4 to 23.0, and 18.3 to 29.3 percentage points in each age group. Improvements were largely due to reductions in moderate and severe anaemia and occurred where the prevalence of acute malnutrition was stable or increasing. No change in stunting was observed in four of five camps. The replicability of findings across five sites strongly suggests that Nutributter^®^ distribution was associated with a reduction in anaemia, but not stunting, among refugee children in the Horn of Africa. Benefits were not restricted to the 6–23 month target group targeted by the nutrition programme. However, even following this intervention anaemia remained a serious public health problem and additional work to define and evaluate an effective intervention package is warranted.

## Introduction

Nutritional anaemia and stunting continue to be serious, worldwide public health issues among refugee populations who are dependent on food assistance, affecting the health and development of children. Both forms of undernutrition have been frequently documented among emergency-affected populations who face limited livelihood opportunities or access to diversified diets, poor sanitation, recurrent illness and infection, and often use suboptimal infant feeding practices[1–7]. Anaemia and stunting adversely affect physical growth, cognitive development, and immune status [8, 9] and severely stunted children have an even higher mortality risk than moderately wasted children [10]. These adverse outcomes justify a focus on both indicators in emergency contexts and the need for more effective interventions to reduce them [11–13].

Fortified blended foods (e.g. Corn-Soy Blend (CSB)) and lipid-based Ready-to-Use Therapeutic Foods (RUTF) have been used in supplementary and therapeutic feeding programmes to prevent and treat acute malnutrition in young children. However, the increasing availability of novel products and their potential role in preventing different types of undernutrition has expanded the scope of traditional selective feeding programmes [14, 15]. ‘Home fortification’ with products designed to complement the existing diet, such as small quantity Lipid-based Nutrient Supplements (SQ-LNS), is increasingly perceived as an effective method for improving the nutritional status of vulnerable groups [14–19].

Nutributter^®^ (Nutriset, Malaunay, France), is a SQ-LNS designed for ‘home fortification’ and it provides a range of vitamins and minerals as well as energy, protein, and essential fatty acids, differentiating it from multiple micronutrient powders and tablets. It is made from peanuts, sugar, vegetable fat, skimmed milk powder, and vitamin and mineral fortificant. Nutributter^®^ is intended to fortify the diets of children aged 6–23 months in contexts where local foods fail to meet their nutritional needs. Early efficacy trials have described some improvement in linear growth and haemoglobin levels after six months of use in infants aged 6–11 months [20, 21]. However, evidence of its effectiveness remains limited to one study in Bangladesh and results from other contexts are difficult to assess as data from control groups is not available and other programs that may impact the measured indicators are often implemented in parallel [22, 23].

In 2008, the United Nations High Commissioner for Refugees (UNHCR) developed and commenced implementation of a strategy aiming to tackle the multiple causes of anaemia and undernutrition in children aged 6–59 months and other vulnerable groups. One of the approaches adopted through this strategy was the introduction of novel, special nutritional products, including Nutributter^®^, distributed (where applicable) through Blanket Supplementary Feeding Programmes (BSFP). This was implemented as part of an integrated strategy to prevent and treat different types of undernutrition [24].

In response to the prevalence of anaemia (haemoglobin <11.0 g/dL) in children 6–59 months being consistently above 60% (the World Health Organization (WHO) threshold indicating anaemia of high public health significance is 40% [25]) Nutributter^®^ distribution was rolled out between 2009–2011 in camps in the East and Horn of Africa. The intervention was implemented in four camps in Kenya (Dagahaley, Hagadera, and Ifo camps in Dadaab, and Kakuma) and one in Djibouti (Ali Addeh). Prior to the intervention, stunting prevalence (height-for-age (HAZ) z-score <-2) was also of poor (20–29%) or serious (30–39%) public health significance in most camps. Between 2008–2009 the prevalence of GAM (weight-for-height z-score <-2) persisted at levels indicating serious/critical public health significance (10 to ≥15%) in all camps, further highlighting the poor nutritional status of vulnerable groups [26]. The aim of this study was to analyse the effectiveness of Nutributter^®^ distribution in reducing anaemia and stunting in refugee children between 2008 and 2011.

## Materials and methods

### Setting and programme context

The Dadaab (Dagahalay, Hagadera, Ifo), and Kakuma refugee camps in Kenya, and the Ali Addeh refugee camp in Djibouti, are all located in semi-arid regions, prone to drought and food insecurity, with limited natural resources and livelihood opportunities. The refugee populations were largely dependent on World Food Programme food assistance, and their diets had low diversity. In Kakuma and Ali Addeh camps, the population doubled during the study period, increasing from 49,500 in 2008 to 84,500 in 2011 in Kakuma, and 8,500 in 2009 to 19,500 in 2011 in Ali Addeh. The combined population of the Dadaab camps in 2008 was 277,000 but rose to at least 483,000 in 2011 due to an influx of refugees fleeing famine in neighbouring Somalia.

From 2008 to 2011 the crude mortality rate and under five mortality rates in all camps were consistently below the Sphere standard of <1.5 deaths/1000/month, and <3deaths/1000/month, respectively (UNHCR Health Information System (HIS)) [27]. From available data, the confirmed average malaria incidence in children aged <5 years in the three camps ranged from 0.33–15.0 cases/1000/month, and was highest in Kakuma, where malaria peaked at >100 cases/1000/month during the 2011 rainy season. Malaria control activities were in place in all camps to varying extents, including bed-net distribution, rapid diagnostic tests, and indoor residual insecticide spraying. Refugees in all five camps had free access to health services, including routine vitamin A and deworming supplementation for children aged 6–59 months and 12–59 months, respectively. Vitamin A supplementation coverage reported in nutrition surveys from 2008 to 2011 was frequently below the >90% target, ranging between 74–97% in Kakuma and Dadaab, and 66% in Ali Addeh in 2011. According to UNHCR’s HIS, the average incidence of parasitic worms diagnosed at health facilities was highest in Ali Addeh at 53.7 cases/1000/month, reaching 120 cases/1000/month in late 2011, compared to 20.6 cases/1000/month and 12.2 cases/1000/months in Dadaab and Kakuma, respectively.

Registered refugees in all camps received a food ration providing at least 2,100 kcal/day, generally consisting of maize meal (210g), wheat flour (400g), corn soy blend (CSB) (50g), pulses (60g), vitamin A and D fortified vegetable oil (30g), sugar (20g) (Ali Addeh only), and salt (5g). CSB plus (CSB with a redesigned micronutrient formulation) replaced CSB around the end of 2010, September 2011, and January 2010 in Dadaab, Kakuma, and Ali Addeh camps, respectively. In Dadaab, families with children 6–12 months received monthly fresh food vouchers (value 1,600 Kenyan Shillings) from September 2009 to 2012. In all camps, complementary foods, including items such as green grams, groundnuts or tuna, were added irregularly to the food basket via the general food distribution or selective feeding programmes.

Children registered in the Nutributter^®^ programme received one 20g sachet of Nutributter^®^ per day that provided 108 kcal of energy and 9mg of elemental iron. The Nutributter^®^ formulation was the standard one supplied by the manufacturer and was similar to formulations used elsewhere [21]. The initial target group for the Nutributter^®^ programme was children 6–23 months, but due to programmatic challenges, the actual target groups in Dadaab and Ali Addeh sometimes differed (Table 1). Children exited the programme when they reached the upper target age limit, or when the programme ended. The Nutributter^®^ interventions were linked to behaviour change communication, growth monitoring and household follow up of defaulters, although the extent to which these activities took place differed by camp. In the Dadaab camps for example, BCC messages were displayed on large posters attached to vans belonging the non-governmental organisation distributing the product.

**Table 1:** Nutributter^®^ distribution protocols and surveys used for assessing the intervention in Dadaab, Kakuma, and Ali Addeh refugee camps.

Camp^1^	Nutributter^®^ distribution dates	Target age group (months)	Duration of distribution (months)	Baseline Survey	End-line survey
Dadaab: Dagahaley, Hagadera, Ifo	Jan 2010 –Mar 2010	12–23	3	Aug 2010	Aug 2011
	Apr 2010 –Jun 2010	12–35	3		
	Dec 2010 –Aug 2011	6–23	9		
Kakuma	Apr 2011 –Oct 2011	6–23	7	Nov 2010	Nov 2011
Ali Addeh^2^	Dec 2009 –Jun 2010	6–23	7	Dec 2008	Oct 2011
	Jul 2010	6–36	1		
	Aug 2010	6–59	1		

^1^ For each camp, the survey immediately before Nutributter^®^ distribution programme began for the recommended target age range of children 6–23 months was selected to use as the baseline. Between January and June 2010, the Dadaab programme targeted a smaller age range than recommended, therefore the next survey after this date was selected to use as the baseline.

^2^ An additional survey was conducted in Ali Addeh in between the baseline and end-line surveys; in March 2010.

### Survey design and sample size

Population representative, cross-sectional surveys were periodically conducted in all camps among children 6–59 months, as part of routine camp health and nutrition monitoring. All surveys used a two-stage cluster sampling methodology, in which the sampling frame for stage one consisted of a list of blocks in the camp, and probability proportional to size was used to sample clusters from these enumeration areas. Households were selected at stage two using the expanded programme for immunisation method or systematic random sampling [28]. Depending on the measures being taken, sub-samples of participants were taken to ensure adequate sample sizes were obtained while maintaining sampling efficiency and minimising respondent burden. For example, for the measurement of haemoglobin during a cluster survey a sub-sample was typically selected by sampling all the eligible children in the first seven households in each cluster, or by measuring haemoglobin in all eligible children in every other household or every third household in each cluster.

From 2008 to 2011, at least one survey per year was conducted in each camp, except in Ali Addeh where no survey was implemented in 2009. For the purpose of this analysis, baseline data for each camp was taken from the survey preceding the start of Nutributter^®^ distribution to the recommended 6–23 month target group (Table 1). In Dadaab, the first Nutributter^®^ distribution ran from January-June 2010 but excluded children 6–12 months, due to a simultaneous food voucher distribution that targeted this age group. Therefore, the baseline was taken as August 2010, preceding the start of distribution to the 6–23 months target group. In the Dadaab and Kakuma camps, the survey data prior to the intervention baseline indicated that the prevalence of anaemia and global acute malnutrition (weight-for-height <-2 z-scores WHO 2006 growth standards and/or nutritional oedema) were relatively stable, and that the selected baseline was therefore representative of the preceding children’s nutritional status. The prevalence of GAM from 2008 to 2011 is presented here as contextual information on the general nutrition and health situation. Nutributter^®^ was distributed as a blanket programme to all eligible children in the camps. There was no control group available for analysis as all children within the target age range in each camp were eligible to receive Nutributter^®^.

All surveys followed standard nutrition survey procedures and the 2011 surveys were implemented in compliance with UNHCR’s newly developed Standardised Expanded Nutrition Survey (SENS) for Refugee Populations guidelines [29]. For each survey, sample size calculations were based on the expected prevalence of GAM in children 6–59 months (based on the previous survey results and adjusted for any expected changes in nutritional status), the desired precision (typically +/- 3–5%), and, for cluster surveys, the expected design effect (typically 1.5 to 2.0). Sample sizes were calculated using Emergency Nutrition Assessment (ENA) for SMART (Standardized Monitoring and Assessment of Relief and Transitions) software [28]. In some surveys haemoglobin was measured in all sampled children, whereas in others, this measurement was done in a randomly selected sub-sample.

### Data collection

In all surveys, enumerators were trained for a minimum of three days including a pilot test. Standardisation activities for anthropometry and testing of Hb were reported for some of the surveys, prior to data collection. Teams were supervised during data collection by survey consultants or nutritionists from UN or NGO agencies working in the camps. Both household and individual level questionnaires were administered during data collection to provide information on a range of indicators. Household level data is not presented here. All surveys used paper questionnaires for data collection, except the Kakuma 2011 survey, which used mobile phones running Open Data Kit [30] on the Android operating system. Data on absences or refusals were not available.

### Measurements

In all surveys, Hb concentration was measured in a capillary blood sample taken from the finger according to standard procedures and analysed using a portable HemoCue 201 or 301 analyser [31]. Anaemia in children aged 6–59 months was defined according to WHO classifications as severe (Hb<7.0g/dL), moderate (Hb <10.0g/dL and ≥7.0g/dL), or mild (Hb <11.0g/dL and ≥10.0g/dL) [25]. All camps were located below 1,000 meters so no adjustment for altitude was made. In more recent surveys, weight was measured using electronic weighing scales to the nearest 100 grams, however in earlier years, hanging spring scales were generally used. Not all survey reports specified the measurement equipment that was used. Length (for children <24 months or <85cm or <87cm) and height (≥24 months or ≥85cm or ≥87cm) was measured using a wooden height board to the nearest mm. Age (day, month, and year) was recorded from either a child health card or birth notification if available. If no reliable proof of age was available, age was estimated in months using a local event calendar. Where age was unknown, only children between 65 and 110 cm were included in the surveys. In the analysis reported here, however, any child with missing age data was excluded.

### Contextual analysis

To aid interpretation of the survey data, additional data were collated, where available, on the following: a) Nutributter^®^ programme monitoring data; b) camp health and nutrition surveillance data; and c) other anaemia prevention and control activities (not presented here). Examples of other anaemia prevention and control activities implemented were as follows: introduction of CSB+ for all age groups via the General Food Ration (all camps); complementary foods added to the food basket for all age groups including tuna fish (Ali Addeh), beans (Ali Addeh), green grams (Dadaab and Kakuma), groundnuts (Dadaab and Kakuma); monthly fresh voucher programme targeted at children 6–12 months (Dadaab); iron supplementation to severely anaemic children (all camps); bed-net distribution (all camps); In-door Residual Spraying campaigns (Dadaab and Kakuma); multi-storey or kitchen gardens for some households (all camps). Limited Nutributter^®^ programme coverage data and adherence data were available. However, a 3 week acceptability test for 120 individuals conducted in 2009 in Ali Addeh [32], and pilot tests conducted in Dadaab and Kakuma prior to the intervention confirmed that the product was well accepted.

### Data analysis

Raw survey data was available in Excel files from 2008 to 2011, but was not available for the Dadaab February 2008 survey and the Kakuma April 2008 survey. In these cases the prevalence estimates reported in the survey reports were used. Cluster numbers were missing from the Ali Addeh, December 2008, baseline survey data file so the confidence intervals reported here for this survey do not allow for the design effect of the cluster sampling.

Anthropometric indices were cleaned and calculated using ENA for SMART software, version July 31^st^ 2012. Indices were calculated using the 2006 WHO Growth Standards [33] and data was usually cleaned using the default ENA nutrition indices cleaning criterion, which uses a flexible inclusion window of +/-3 reference z-scores from the observed mean. However, data from the Dadaab surveys in 2011 was available in a form that that had already been cleaned using an inclusion window of +/-4 reference z-scores for weight-for-height, due to the unusually high prevalence of acute malnutrition, and was not re-cleaned prior to analysis. In both cases, records with anthropometric z-scores that fell outside of the designated range were dropped from analysis.

Statistical analysis of anaemia, haemoglobin concentration, and stunting followed a pre-post design and was based on intention to treat, regardless of intake. Analysis was conducted on two different age groups: 6–59 months (standard age group assessed in emergency nutrition surveys) and 6–23 months (age group Nutributter^®^ was designed for). Initially, trends in anaemia, haemoglobin, and stunting levels were graphed and visually reviewed. For surveys conducted in the Kakuma and Dadaab camps, differences between baseline and end-line surveys were then tested for using logistic and linear regression, using the svy command in Stata version 14 and accounting for the clustering of the data. All regression models were adjusted for age due to differences in the age distributions between surveys and the correlation seen between age and Hb concentration. Regression analysis was not possible for the Ali Addeh camp due to the missing cluster numbers. P-values of <0.05 was considered statistically significant.

### Ethics

Ethical permission was not required as the analysis was of routinely collected and anonymised cross-sectional data. The data was entered in to electronic databases without any personal identifiers. No medical data were collected prospectively for this study and the surveys were performed before the research began. The authors did not have access to personal information of the participants such as names, or birth dates before they had access to the study data. Finger stick blood samples have been routinely collected in UNHCR nutrition surveys for some years and their collection was not linked to, or took place because of the subsequent research. Some of the authors were involved in some of the nutrition surveys as part of their technical support or survey coordination work.

Participants did not provide written consent to having their data used for research purposes as this was routinely collected data and was not intended for research purposes. Verbal consent was requested from survey participants as is required by standard survey practice and was recorded on a paper list or in a field on the questionnaire. It was made clear that any entitlements to humanitarian or other forms of assistance would not be affected by their decision to participate or not. Parental or carer consent was obtained for all participating minors.

## Results

The planned sample size for measuring anaemia in children aged 6–59 months ranged from 252 to 531 across all camps and years, while the achieved sample ranged from 236 to 620 children. All but one of the baseline and end-line surveys exceeded the planned sample size. Sample sizes for the anthropometric measurement of stunting ranged from 305–604 children. Proof of age documentation was reported in Kakuma as 91% in 2011, but ranged from 60–88% in previous years. The remaining camps only reported on proof of age documentations in 2011, when it ranged from 36–55%. There was a significant difference in mean reported age between the baseline (2010) and end-line (2011) surveys in Dadaab and Kakuma camps (p<0.05), but not in Ali Addeh.

### Graphical analysis

**Fig 1:**
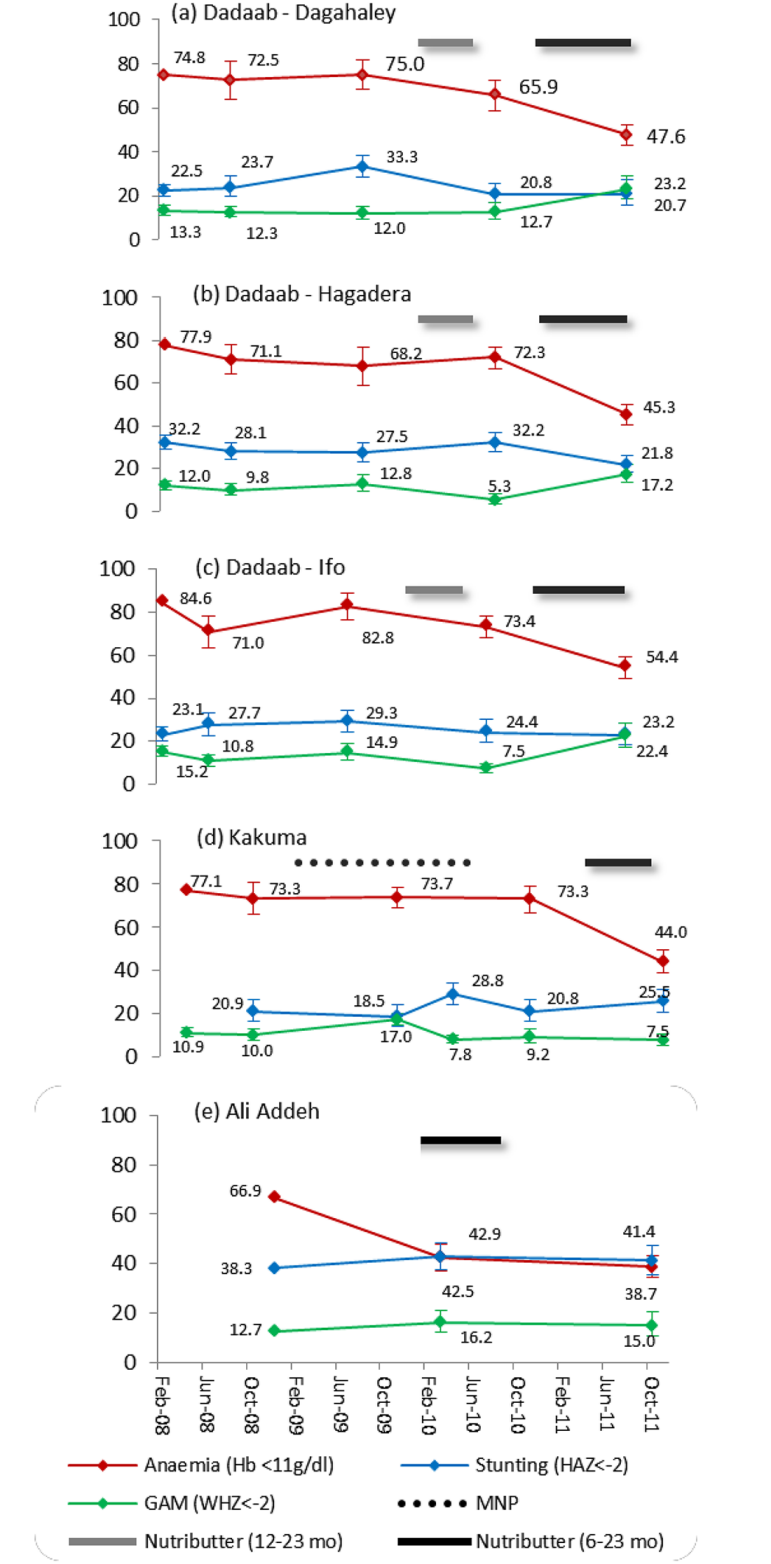
Anaemia, stunting, and global acute malnutrition (GAM) prevalence in children aged 6–59 months between 2008 and 2011.

Graphing of standard programme indicators (anaemia, stunting, and GAM in children 6–59 months) for the years prior to and including the baseline surveys revealed that the levels of anaemia were very high (>66%) and stable in all camps. However, they appeared to fall markedly after the introduction of the Nutributter^®^ programme in four out of the five camps (Dadaab-Dagahaley, Dadaab-Hagadera, Dadaab-Ifo, Kakuma) (Fig 1). In Dadaab-Hagadera, a significant anaemia decrease was only seen following the second distribution programme. In Ali Addeh, the decrease in anaemia levels was not as large as in the other camps after the introduction of the product. In contrast, the prevalence of stunting within the camps remained relatively stable between 2008 and 2011, except for a decrease in Dadaab-Hagadera camp. The prevalence of GAM also appeared to change little except when it rose steeply in the Dadaab camps in 2011, an increase associated with the famine in Southern Somalia and an influx of new refugees.

**Fig 2:**
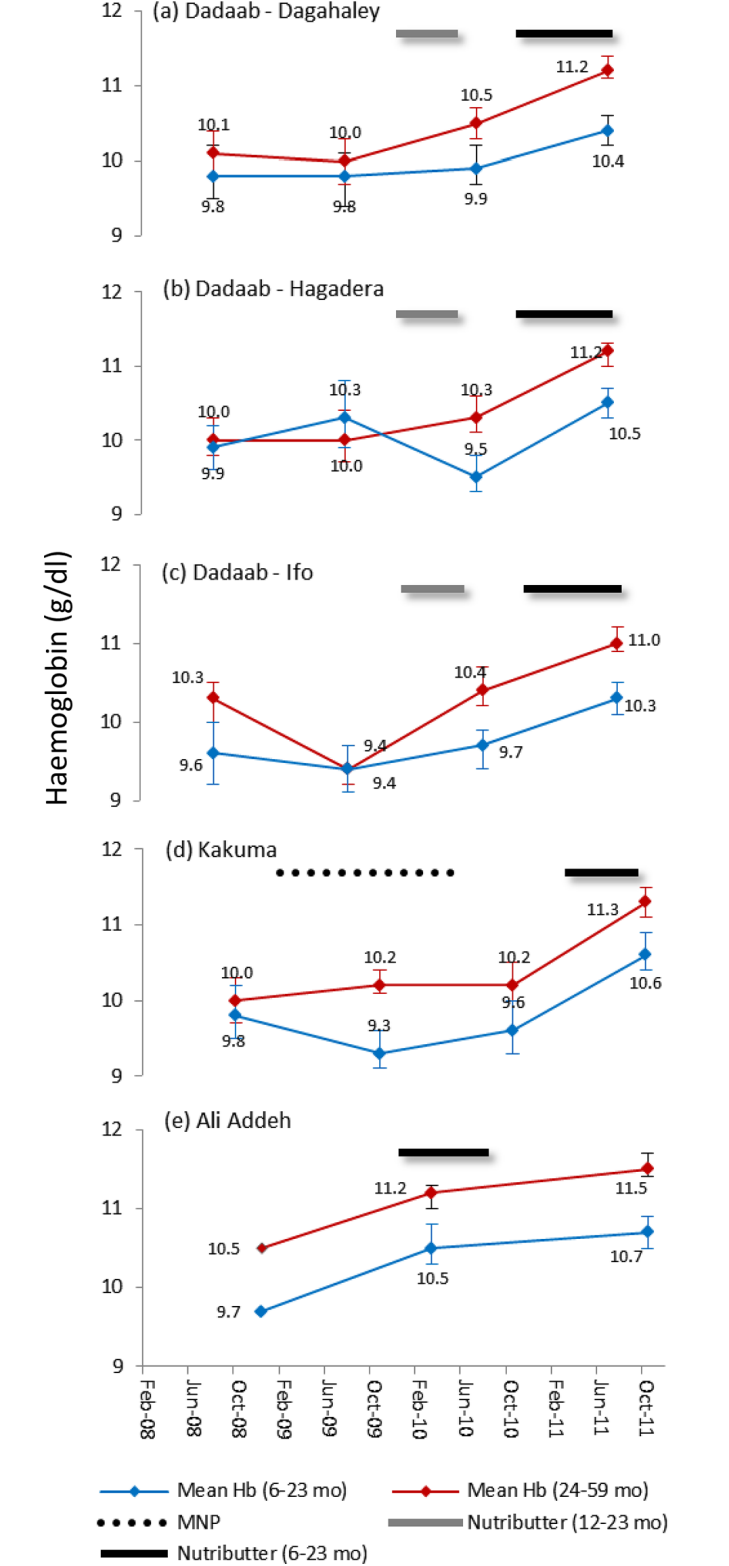
Mean haemoglobin concentration in children aged 6–23 and 24–59 months between 2008 and 2011.

In Fig 2. the mean haemoglobin concentration is presented for the same period, both for the 6–23 month age group that was targeted to receive the Nutributter^®^ distribution, and for children aged 24–59 months. In all camps and both age groups, haemoglobin concentration is observed to increase following the Nutributter^®^ distribution, except for a decline in children aged 6–23 months in Dadaab-Hagadera following the first distribution programme. In Kakuma camp no increase in haemoglobin is observed following the blanket distribution of a low iron dose (2.5 mg) micronutrient powder that began in 2008, which may reflect the relatively low acceptability, adherence, or efficacy of this product [34]. To test for significant changes in anaemia and stunting, regression analysis was conducted to allow for the difference in ages between the baseline and end-line survey samples to be adjusted for.

### Anaemia and haemoglobin concentration

Table 2 presents regression analysis of haemoglobin concentration and anaemia for the 6–23 month age group that was the target of the intervention. Following the intervention, the mean haemoglobin concentration in the target group increased in all camps where statistical analysis was possible, rising by between 0.47 to 0.98 g/dL. In Ali Addeh, the mean haemoglobin concentration was 9.7 g/dL at baseline and 10.7 g/dL at end-line, a difference comparable to that observed in other camps.

**Table 2:** Regression analysis of mean haemoglobin and the prevalence of anaemia at baseline and end-line in children aged 6–23 months.

Camp	Time point	Haemoglobin (g/dL)	Difference (g/dL)^1^	p	Anaemia % (<11g/dL)	Odds ratio	P
Dagahaley	Baseline (n = 233)	9.9 (9.9, 10.2)	0.47 (0.19, 0.75)	<0.01	72.1 (64.7, 78.5)	0.65 (0.41, 1.02)	0.06
	End-line (n = 180)	10.4 (10.2, 10.6)			62.8 (55.3, 69.7)		
Hagadera	Baseline (n = 229)	9.5 (9.3, 9.8)	0.94 (0.60, 1.28)	<0.01	82.1 (76.8, 86.4)	0.40 (0.25, 0.62)	<0.01
	End-line (n = 205)	10.5 (10.3, 10.7)			63.4 (55.9, 70.3)		
Ifo	Baseline (n = 279)	9.7 (9.4, 1.0)	0.63 (0.27, 1.00)	<0.01	79.2 (73.6, 83.9)	0.52 (0.32, 0.84)	0.01
	End-line (n = 184)	10.3 (10.1, 10.5)			66.8 (58.3, 74.4)		
Kakuma	Baseline (n = 117)	9.6 (9.3, 10.0)	0.98 (0.55, 1.41)	<0.01	80.3 (69.5, 88.0)	0.33 (0.17, 0.64)	<0.01
	End-line (n = 225)	10.6 (10.4, 10.9)			57.3 (49.3, 65.0)		
Ali Addeh^2^	Baseline (n = 103)	9.7 (9.5, 10.0)	N/A	N/A	78.6 (69.5, 85.6)	N/A	N/A
	End-line (n = 187)	10.7 (10.5, 10.9)			55.6 (48.3, 62.7)		

^1^ The values for the difference in mean Hb are linear regression coefficients (95% confidence interval), and for comparison of anaemia prevalence odds ratios (95% CI) are given. Both analyses account for clustering and are adjusted for age. Linear regression coefficients represent the increase in mean haemoglobin between baseline and end-line. The adjusted odds ratio represents the odds of having anaemia at end-line compared to the odds at baseline.

^2^ Regression analyses from Ali Addeh are not included as it was not possible to account for clustering due to missing data.

The OR presented in Table 2 also indicate a significant reduction in the prevalence of anaemia in 3 of the 4 camps analysed (with borderline significance in Dagahaley). The magnitude of reduction ranged between 12.9 and 23.0 percentage points. Results in Ali Addeh were comparable to that seen in Kakuma although it was not possible to assess if the difference was significant.

To assess the effects of Nutributter^®^ distribution on the standard age group that is assessed in emergency nutrition surveys, we also looked at the changes in the combined 6–59 month age group. In this age group, the mean haemoglobin concentration increased significantly in all of the analysed camps following intervention (Table 3). In the years prior to the intervention, anaemia prevalence in children aged 6–59 months ranged from 65.9% to 84.6% (Fig 2). Following the intervention, the prevalence of anaemia decreased significantly in all camps compared to baseline, with relative reductions (i.e. reduction as a percentage of the baseline) ranging from 26–42%. Surveys of the Dadaab camps in 2011 indicated that anaemia prevalence had significantly decreased despite the significant increase in the prevalence of GAM in children aged 6–59 months.

**Table 3:** Regression analysis of mean haemoglobin and the prevalence of anaemia at baseline and end-line in children aged 6–59 months.

Camp	Time point	Haemoglobin (g/dL)	Difference (g/dL)^1^	p	Anaemia % (<11g/dL)	Odds ratio	P
Dagahaley	Baseline (n = 437)	10.2 (10.0, 10.4)	0.60 (0.36, 0.85)	<0.01	65.9 (58.4, 72.7)	0.53 (0.36, 0.76)	<0.01
	End-line (n = 576)	10.9 (10.8, 11.1)			47.6 (43.1, 52.1)		
Hagadera	Baseline (n = 441)	9.9 (9.7, 10.2)	0.90 (0.63, 1.117)	<0.01	72.3 (67.0, 77.1)	0.35 (0.25, 0.50)	<0.01
	End-line (n = 598)	11.0 (10.8, 11.1)			45.3 (40.6, 50.1)		
Ifo	Baseline (n = 433)	9.9 (9.7, 10.2)	0.58 (0.30, 0.87)	<0.01	73.4 (68.0, 78.2)	0.55 (0.38, 0.80)	<0.01
	End-line (n = 550)	10.7 (10.6, 10.9)			54.4 (49.6, 59.1)		
Kakuma	Baseline (n = 236)	9.9 (9.7, 10.2)	1.05 (0.73, 1.37)	<0.01	73.3 (66.4, 79.2)	0.30 (0.20, 0.46)	<0.01
	End-line (n = 620)	11.1 (10.9, 11.3)			44.0 (38.7, 49.5)		
Ali Addeh^2^	Baseline (n = 314)	10.3 (10.1, 10.4)	N/A	N/A	66.9 (61.5, 71.9)	N/A	N/A
	End-line (n = 563)	11.2 (11.1, 11.4)			38.7 (34.7, 43.0)		

^1^ The values for the difference in mean Hb are linear regression coefficients (95% confidence interval), and for comparison of anaemia prevalence odds ratios (95% CI) are given. Both analyses account for clustering and are adjusted for age. Linear regression coefficients represent the increase in mean haemoglobin between baseline and end-line. The adjusted odds ratio represents the odds of having anaemia at end-line compared to the odds at baseline.

^2^ Regression analyses from Ali Addeh are not included as it was not possible to account for clustering due to missing data.

### Anaemia severity

In all five camps and in both age groups studied, improvements in anaemia were largely attributable to reductions in moderate and severe anaemia (S1 Table). In children aged 6–23 months in both Kakuma and Ali Addeh, moderate anaemia was reduced by at least half at end-line compared to baseline. Severe anaemia was no longer observed at end-line in children aged 6–23 months in Dadaab-Hagadera and Ali Addeh. In children 6–59 months, moderate anaemia approximately halved between baseline and end-line in all camps (p<0.05). The number of children with severe anaemia in Dagahaley, Hagadera, Ifo, Kakuma, and Ali Addeh was, respectively, 10, 20, 18, 6 and 9 at baseline and 2, 3, 5, 5, and 2 at end-line.

### Stunting

The prevalence of stunting was comparable for children aged 6–23 months and those aged 6–59 months in all camps, and showed little indication of change following the intervention (Tables 4 and 5). In children 6–23 months, stunting decreased significantly between baseline and end-line only in Dadaab-Hagadera. In Dadaab-Dagahaley and Dadaab-Ifo, stunting remained unchanged between baseline and end-line. However, there was some variation in the prevalence of stunting in the years prior to the Nutributter^®^ distribution (Fig 1). For example, a spike in prevalence was seen in Dagahaley in 2009 followed by a sharp reduction in 2010. Similar variation was seen in Kakuma where stunting was <20% in 2008 and 2009, but doubled in April 2010 rising from 14.1% (95% CI 9.5–20.5) in 2009 to 30.2% (95% CI 25.3–35.6). In Ali Addeh the prevalence of stunting remained stable but relatively high across all surveys. The pattern observed for stunting in children aged 6–59 months was similar to that observed in the younger sub-group.

**Table 4:** Regression analysis of mean height for age and stunting prevalence (HAZ<-2) in children aged 6–23 months.

Camp	Time point	Height-for-age z-score	Difference^1^	p	Stunting (%) HAZ <-2	Odds ratio	P
Dagahaley	Baseline (n = 267)	-1.10 (-1.28, -0.93)	0.21 (-0.08, 0.51)	0.10	20.2 (15.2, 26.5)	0.88 (0.46, 1.71)	0.70
	End-line (n = 162)	-0.88 (-1.11, -0.64)			17.9 (11.2, 27.4)		
Hagadera	Baseline (n = 300)	-1.27 (-1.41, -1.13)	0.28 (0.03, 0.54)	0.03	28.7 (24.3, 33.5)	0.63 (0.41, 0.97)	0.04
	End-line (n = 189)	-0.92 (-1.13, -0.70)			19.0 (14.3, 24.9)		
Ifo	Baseline (n = 314)	-0.93 (-1.13, -0.74)	-0.16 (-0.47, 0.16)	0.30	22.3 (17.5, 27.9)	1.03 (0.60, 1.78)	0.90
	End-line (n = 170)	-1.08 (-1.34, -0.82)			22.4 (15.4, 31.2)		
Kakuma	Baseline (n = 262)	-0.83 (-1.09, -0.57)	-0.29 (-0.62, 0.03)	0.08	18.3 (12.6, 25.9)	1.61 (0.89, 2.91)	0.10
	End-line (n = 220)	-1.13 (-1.33, -0.92)			26.4 (19.6, 34.4)		
Ali Addeh^2^	Baseline (n = 101)	-1.89 (-2.13, -1.65)	N/A	N/A	42.6 (33.2, 52.5)	N/A	N/A
	End-line (n = 167)	-1.64 (-1.88, -1.40)			40.1 (30.2, 50.9)		

^1^ The values for the difference in mean HAZ are linear regression coefficients (95% confidence interval), and for comparison of stunting prevalence odds ratios (95% CI) are given. Both analyses account for clustering and are adjusted for age. Linear regression coefficients represent the change in mean HAZ between baseline and end-line. The adjusted odds ratio represents the odds of being stunted at end-line compared to the odds at baseline.

^2^ Regression analyses from Ali Addeh are not included as it was not possible to account for clustering due to missing data.

**Table 5:** Regression analysis of mean height for age and stunting prevalence (HAZ<-2) in children aged 6–59 months.

Camp	Time point	Height-for-age z-score	Difference^1^	p	Stunting (%) HAZ <-2	Odds ratio	p
Dagahaley	Baseline (n = 520)	-1.09 (-1.25, -0.93)	0.14 (-0.10, 0.37)	0.2	20.8 (16.8, 25.4)	0.98 (0.63, 1.54)	0.9
	End-line (n = 537)	-0.95 (-1.11, -0.78)			20.7 (15.4, 27.1)		
Hagadera	Baseline (n = 600)	-1.43 (-1.57, -1.30)	0.44 (0.25, 0.63)	<0.01	31.8 (27.5, 36.5)	0.58 (0.43, 0.80)	<0.01
	End-line (n = 574)	-1.02 (-1.16, -0.89)			21.8 (18.1, 26.0)		
Ifo	Baseline (n = 516)	-1.05 (-1.22, -0.87)	-0.02 (-0.27, 0.23)	0.9	24.4 (19.4, 30.2)	0.92 (0.59, 1.43)	0.7
	End-line (n = 504)	-1.08 (-1.25, -0.92)			23.2 (18.5, 28.7)		
Kakuma	Baseline (n = 559)	-0.92 (-1.13, -0.71)	-0.19 (-0.46, 0.07)	0.2	20.8 (16.1, 26.3)	1.32 (0.87, 1.99)	0.2
	End-line (n = 604)	-1.11 (-1.28, -0.95)			25.5 (20.8, 30.9)		
Ali Addeh^2^	Baseline (n = 305)	-1.83 (-1.97, -1.69)	N/A	N/A	42.6 (33.2, 52.5)	N/A	N/A
	End-line (n = 512)	-1.74 (-1.88, -1.59)			41.4 (35.5, 47.6)		

^1^ The values for the difference in mean HAZ are linear regression coefficients (95% confidence interval), and for comparison of stunting prevalence odds ratios (95% confidence interval) are given. Both analyses account for clustering and are adjusted for age. Linear regression coefficients represent the change in mean HAZ between baseline and end-line. The adjusted odds ratio represents the odds of being stunted at end-line compared to the odds at baseline.

^2^ Regression analyses from Ali Addeh are not included as it was not possible to account for clustering due to missing data.

## Discussion

The secondary data analysis reported here suggests that Nutributter^®^ distribution is effective at reducing anaemia in pre-school children in refugee camp settings in east Africa. While our study design used a before and after analysis of routinely collected household survey data, the overall consistency of findings in five camps from three different refugee contexts lends weight to our findings. Changes in the prevalence of stunting were negligible and, as expected, the prevalence of acute malnutrition did not decrease in response to the intervention. To our knowledge, this article is one of the first assessments of the effectiveness of a SQ-LNS in reducing anaemia and stunting in refugee children between 6–59 months and could have implications for other contexts and geographical regions.

Following the intervention, mean haemoglobin levels increased significantly in children 6–23 months in all camps, and the prevalence of anaemia reduced significantly in four of the five camps. In all camps, anaemia prevalence was lower and haemoglobin concentration higher in the older children. This was to be expected because iron requirements are highest in children 6–12 months of age [35] and older children may have better access to a wider variety of foods. In children aged 6–59 months, significant reductions in anaemia were seen in all camps exceeding the UNHCR short-term target of at least 20% relative reduction in anaemia [11]. In fact, Kakuma and Ali Addeh camps achieved considerably more than this with ≥40% relative reductions. This finding indicates that the benefits of the intervention may not have been restricted to the target group. Factors contributing to improvements outside of the target group are likely to have included the sharing of Nutributter^®^ with older children at household level, which was anecdotally reported, as well as exposure to other interventions. In addition, some older children who were included in the surveys would have been eligible to receive Nutributter^®^ in the recent past.

The reduction in anaemia in all camps and both age groups was largely accounted for by reductions in moderate and severe anaemia, with moderate anaemia halving between baseline and end-line. Mild anaemia remained relatively constant during the intervention period in children aged 6–59 months, but showed a tendency to increase in the younger sub-group, presumably due to the shift in the distribution curve of the population that resulted in less moderate and severe cases. The lack of reduction in the prevalence of mild anaemia suggests that other modalities may be needed to treat mild anaemia or that the current diagnostic cut-off may require adjustment for African populations [36].

Results suggest that improvements in anaemia might be possible after as little as three months of Nutributter^®^ distribution and even sustained over a period after distribution is halted. In Ali Addeh, anaemia prevalence between baseline (2008) and midline (2010) reduced by 19% and 28% in children aged 6–23 months and aged 6–59 months respectively (p-value not available), despite the Nutributter^®^ programme having started just three months prior to a midline survey. Anaemia prevalence was sustained by the end-line survey which occurred 13 months after the Nutributter^®^ programme had ceased. However, other contextual or programmatic factors are likely to have contributed to these improvements in Ali Addeh, and it also has to be considered that the baseline survey commenced 12 months prior to the intervention starting, so it may not have reflected the population status immediately before the Nutributter^®^ programme began. These findings are plausible however, as evidence indicates that a response to iron supplementation is likely within 3 months [37], although reductions in anaemia after consumption of SQ-LNS have, until now, only been documented in children after 6–9 months of use [21, 38]. Nevertheless, whilst causality cannot be confirmed, these findings warrant further investigation as to whether short distribution cycles during vulnerable periods may still be beneficial in reducing anaemia, bearing in mind that it takes time for programmes to become established and used effectively by communities.

Despite these considerable gains, anaemia prevalence at end-line in children aged 6–23 months persisted above 55% in all camps. In children aged 6–59 months, anaemia decreased below 40% (WHO threshold for high public health significance) in Ali Addeh only. This persistently high anaemia prevalence, after seven to nine months of Nutributter^®^ distribution raises important questions relevant to future programming. Evidence from other contexts (non-refugee) similarly found that some anaemia remained after consumption of SQ-LNS, [21, 38] and that this is likely to be due to non-nutritional causes or failure to consume the product as recommended. Despite a significant reduction in anaemia prevalence in infants receiving SQ-LNS for 9 months, Hess et al found that 79% of children still had Hb<110g/dL at end-line [38]. Adu-Afarwuah et al found that after six months consumption of Nutributter^®^ by children from six months of age, some anaemia still remained, and suggested that that this may be due to other infectious or other non-nutritional factors [21]. Gera et al [37] also highlighted the effect of non-nutritional factors on anaemia levels. The authors reviewed the effects of iron supplementation on haemoglobin concentration in children and found that between 38–62% of baseline anaemia (<110g/dL) was due to iron deficiency and responsive to iron supplementation in non-hyperendemic malarial areas, compared to 6–32% in hyperendemic areas. Whilst haemoglobin concentration can be improved with use of fortified lipid-based products, anaemia due to non-nutritional factors may be limiting further reductions. Other non-diet related causes including haemoglobinopathies, thalassemia, malaria, intestinal worms, and the ‘anaemia of inflammation’ need to be considered in efforts to improve anaemia rates.

Nutributter^®^ appeared to have little effect on the prevalence of stunting. Significant changes in stunting between baseline and end-line were only seen in Dadaab-Hagadera, where decreases were seen in both of the studied age groups. The reasons for the differential response in this camp are unclear. Possible reasons include that children’s exposure to the intervention may have been less than 6 months, depending on their age when the distribution period began and ended. Furthermore, children who were already 12–23 months of age when the distribution began would have had no exposure during the critical period between 6 and 12 months, when a response in linear growth is most likely. Stunting levels were also fairly low at <30% (classified as poor according WHO standards [26]) in 16 of 23 surveys between 2008 and 2011 for which data was available. As a result, small changes would be difficult to detect through routine cross-sectional surveys with limited sample sizes and relatively low prevalence estimates.

Evidence from early trials found that severe stunting (LAZ<-3) occurred less often in six month old children receiving 25-50g of LNS (with a similar nutrient content to Nutributter^®^) for one year, compared to standard porridge, and that effects were most pronounced in children who had a low LAZ to start with [39, 40]. More recent studies using 25-50g LNS for the prevention of malnutrition have found mixed effects. Three trials in Burkina Faso, Haiti, and Ghana using SQ-LNS have reported positive effects on stunting and /or linear growth,[38, 41, 42] although questions have been raised over the findings of the Haiti trial [43]. However three studies in Malawi [44–46] did not support this finding, suggesting that positive effects in other trials may be due to initial differences in length-for-age, contextual factors, and the combination of interventions provided. The lack of association of Nutributter^®^ with stunting reduction in this study underlines the need to pursue multi-sectoral stunting reduction interventions in refugee contexts as well as in development work.

This study has a number of important limitations. Firstly, since this was a pre-post assessment using routine cross-sectional survey data, representative of children aged 6–59 months, there was no control group. There were changes in the camp populations during the study’s time periods, which brought about additional variability compared with repeat surveys in more stable refugee communities. Therefore, our data describes a plausible association between the Nutributter^®^ programme and anaemia reduction but is unable to attribute causality with a statistical probability [47]. Proof of age estimates varied greatly (36–91%) and were not reported for all surveys; therefore the accuracy of stunting estimates in some surveys may be lower due to poor age estimations. Z-scores for height-for-age require accurate ages to within two weeks [48].

The available surveys did not always occur immediately before or after the Nutributter^®^ distributions, and the programme target groups varied, particularly in Dadaab, posing challenges to the interpretation of results. The results presented here also need to be interpreted in consideration of the other activities implemented concurrently as part of UNHCR’s anaemia strategy from 2008 to 2011. All camps received complementary foods contributing to improved dietary diversity. A number of activities were reported to have been initiated or strengthened as a result of the anaemia strategy such as routine vitamin A supplementation and de-worming campaigns, vector control activities (particularly in Kakuma), and iron supplementation to pregnant women. The introduction of improved CSB (i.e. Super Cereal) to the general ration in all five camps during 2010–2011 may also have contributed to reductions in anaemia [49]. Unfortunately, despite the best efforts of the researchers it was not possible to confirm the exact implementation dates for these activities, nor to what extent a combination of these interventions were consistently implemented over a specific time period.

A further challenge in evaluating the effectiveness of the Nutributter^®^ programme was the lack of adherence monitoring data, both for the intervention itself and other anaemia strategy activities. Reported adherence from studies providing daily SQ-LNS have been high [38, 44], although adherence recorded through home observations maybe somewhat lower [38, 50]. Evidence from other contexts also indicates that the product is well accepted [21, 51–55]. However, the high acceptability of the product may also lead to greater sharing with other children or adults and a lower intake by the target group [55].

Despite limitations in the study design, the strength of evidence of this analysis lies in the strikingly similar findings of reduced anaemia prevalence found in five camps in three different refugee contexts, following the distribution of Nutributter^®^. Given the constraints described above, replicability of impact in different contexts has been highlighted as key to the validation of such interventions [56].

This analysis therefore supports the use of Nutributter^®^ as an effective means of reducing anaemia in certain refugee populations, as part of a multi-sectoral approach. The growing issue of the double burden of under nutrition and obesity in some emergency affected and refugee populations should be considered before implementing the use of LNS products [57].

Further efforts are needed to reduce anaemia and stunting in young children as malnutrition suffered in early life can lead to permanent and potentially generational impairment [58]. The development of public health thresholds for the prevalence of anaemia and anthropometric indicators in children 6–23 months would aid the interpretation of data from this younger age group. There is a need for more studies on the effectiveness of SQ-LNS, such as Nutributter^®^, for reducing anaemia and stunting in other settings, as well as on their cost-effectiveness compared to other approaches and products.

## Supporting information

S1 Table Prevalence of anaemia categories at baseline and end-line in children aged 6–23 months in Dadaab, Kakuma, and Ali Addeh refugee camps. (DOCX)

S2 Table Prevalence of anaemia categories at baseline and end-line in children aged 6–59 months in Dadaab, Kakuma, and Ali Addeh refugee camps. (DOCX)

S3 Table Stunting prevalence (HAZ<-2, HAZ<-3) in children aged 6–23 months. (DOCX)

S4 Table Stunting prevalence (HAZ<-2, HAZ<-3) in children aged 6–59 months. (DOCX)

S1 Questionnaire 6-59-months-Dadaab-2011. (DOC)

## Abbreviations

BCC: behaviour change communication
CI: confidence interval
CSB: corn soy blend
GAM: global acute malnutrition
HAZ: height-for-age z-score
Hb: haemoglobin
HIS: Health Information System
NGO: nongovernmental organisation
OR: odds ratio
RUTF: ready to use therapeutic food
SQ-LNS: small quantity lipid nutrient supplement
UNHCR: United Nations High Commissioner for Refugees
WHO: World Health Organization
