# Supplementary material for: Assessment of the effectiveness of a small quantity lipid-based nutrient supplement on reducing anaemia and stunting in refugee populations in the Horn of Africa: Secondary data analysis
Source: PLoS One. 2017 Jun 7;12(6):e0177556. doi: 10.1371/journal.pone.0177556 (PMC5462343; doi:10.1371/journal.pone.0177556)
Supplement: S1 Table — (DOCX) [file pone.0177556.s001.docx]

**S1 Table. Prevalence of anaemia categories at baseline and end-line in children aged 6-23 months in Dadaab, Kakuma, and Ali Addeh refugee camps^1^**

| **Camp** | **Time point** | **Mild  (10.0-10.9 g/dl)** | **Moderate  (7.0-9.9 g/dl)** | **Severe  (<7.0 g/dl)** |
| --- | --- | --- | --- | --- |
| Dagahaley | Baseline (n=233) | 24.5 (19.1, 30.8) | 45.1 (37.4, 53.0) | 2.6 (0.94, 6.84) |
|  | End-line (n=180) | 29.4 (22.7, 37.3) | 32.2 (24.7, 40.7) | 1.1 (0.27, 4.42) |
| Hagadera | Baseline (n=229) | 27.5 (21.7, 34.2) | 48.9 (41.8, 56.1) | 5.7 (2.57, 12.1) |
|  | End-line (n=205) | 32.2 (26.2, 38.9) | 31.2 (25.0, 38.2) | 0.00 |
| Ifo | Baseline (n=279) | 25.8 (21.1, 31.1) | 48.0 (41.2, 54.9) | 5.4 (2.64, 10.7) |
|  | End-line (n=184) | 29.3 (22.0, 37.9) | 37.0 (28.7, 46.1) | 0.5 (0.07, 3.94) |
| Kakuma | Baseline (n=117) | 19.7 (13.4, 27.9) | 59.0 (48.2, 68.9) | 1.7 (0.43, 6.50) |
|  | End-line (n=225) | 31.1 (24.5, 38.6) | 24.9 (19.2, 31.5) | 1.3 (0.32, 5.43) |
| Ali Addeh | Baseline (n=103) | 23.3 (16.0, 32.6) | 52.4 (42.7, 62.0) | 2.9 (0.92: 8.80) |
|  | End-line (n=187) | 31.0 (25.0, 37.7) | 24.6 (18.8, 31.5) | 0.00 |

^1^ The data are prevalence % (95% Confidence Interval). Cluster numbers were not available for the baseline survey in Ali Addeh so confidence intervals were calculated without allowing for clustering.
