# Supplementary material for: Assessment of the effectiveness of a small quantity lipid-based nutrient supplement on reducing anaemia and stunting in refugee populations in the Horn of Africa: Secondary data analysis
Source: PLoS One. 2017 Jun 7;12(6):e0177556. doi: 10.1371/journal.pone.0177556 (PMC5462343; doi:10.1371/journal.pone.0177556)
Supplement: S2 Table — (DOCX) [file pone.0177556.s002.docx]

**S2 Table. Prevalence of anaemia categories at baseline and end-line in children aged 6-59 months in Dadaab, Kakuma, and Ali Addeh refugee camps^1^**

| **Camp** | **Time point** | **Mild  (10.0-10.9 g/dl)** | **Moderate  (7.0-9.9 g/dl)** | **Severe  (<7.0 g/dl)** |
| --- | --- | --- | --- | --- |
| Dagahaley | Baseline (n=437) | 23.3 (19.4, 27.8) | 40.3 (34.2, 46.7) | 2.3 (1.09, 4.73) |
|  | End-line (n=576) | 27.3 (23.4, 31.5) | 20.0 (16.6, 23.8) | 0.4 (0.09, 1.40) |
| Hagadera | Baseline (n=441) | 26.5 (22.3, 31.3) | 41.3 (36.2, 46.5) | 4.5 (2.52, 8.04) |
|  | End-line (n=598) | 24.2 (20.7, 28.2) | 20.6 (17.0, 24.6) | 0.5 (0.16, 1.53) |
| Ifo | Baseline (n=433) | 27.7 (23.6, 32.3) | 41.6 (36.1, 47.2) | 4.2 (2.26, 7.54) |
|  | End-line (n=550) | 27.5 (23.3, 32.1) | 26.0 (21.7, 30.8) | 0.9 (0.39, 2.10) |
| Kakuma | Baseline (n=236) | 23.3 (19.2, 27.9) | 47.5 (40.6, 54.4) | 2.5 (1.19, 5.36) |
|  | End-line (n=620) | 24.8 (21.2, 28.8) | 18.4 (15.0, 22.3) | 0.8 (0.29, 2.21) |
| Ali Addeh | Baseline (n=314) | 27.4 (22.7, 32.6) | 37.6 (32.4, 43.1) | 1.9 (0.86, 4.20) |
|  | End-line (n=563) | 22.4 (19.6, 25.4) | 16.0 (12.7, 20.0) | 0.4 (0.09, 1.45) |

^1^ The data are prevalence % (95% Confidence Interval). Cluster numbers were not available for the baseline survey in Ali Addeh so confidence intervals were calculated without allowing for clustering.
