# Supplementary material for: Assessment of the effectiveness of a small quantity lipid-based nutrient supplement on reducing anaemia and stunting in refugee populations in the Horn of Africa: Secondary data analysis
Source: PLoS One. 2017 Jun 7;12(6):e0177556. doi: 10.1371/journal.pone.0177556 (PMC5462343; doi:10.1371/journal.pone.0177556)
Supplement: S3 Table — (DOCX) [file pone.0177556.s003.docx]

**S3 Table. Stunting prevalence (HAZ<-2, HAZ<-3) in children aged 6-23 months^1^**

| **Camp** | **Time point** | **Height-for-age z-score <-2 and ≥-3** | **Height-for-age z-score <-3** |
| --- | --- | --- | --- |
| Dagahaley | Baseline (n=267) | 13.5 (9.30, 19.2) | 6.7 (4.03, 11.1) |
|  | End-line (n=162) | 13.0 (7.61, 21.2) | 4.9 (2.22, 10.6) |
| Hagadera | Baseline (n=300) | 21.0 (17.6, 24.9) | 7.7 (4.87, 11.9) |
|  | End-line (n=189) | 14.8 (11.0, 19.6) | 4.2 (2.11, 8.32) |
| Ifo | Baseline (n=314) | 17.5 (13.6, 22.2) | 4.8 (2.65, 8.47) |
|  | End-line (n=170) | 15.3 (10.3, 22.2) | 7.6 (3.78, 12.8) |
| Kakuma | Baseline (n=262) | 11.5 (8.13, 15.9) | 6.9 (3.86, 11.9) |
|  | End-line (n=220) | 20.9 (15.8, 27.1) | 5.5 (2.65, 10.9) |
| Ali Addeh | Baseline (n=101) | 22.8 (15.5, 32.1) | 19.8 (13.0, 28.9) |
|  | End-line (n=167) | 26.3 (19.8, 34.2) | 13.8 (8.63, 21.2) |

^1^ The data are prevalence % (95% Confidence Interval). Cluster numbers were not available for the baseline survey in Ali Addeh so confidence intervals were calculated without allowing for clustering.
