# Supplementary material for: Assessment of the effectiveness of a small quantity lipid-based nutrient supplement on reducing anaemia and stunting in refugee populations in the Horn of Africa: Secondary data analysis
Source: PLoS One. 2017 Jun 7;12(6):e0177556. doi: 10.1371/journal.pone.0177556 (PMC5462343; doi:10.1371/journal.pone.0177556)
Supplement: S4 Table — (DOCX) [file pone.0177556.s004.docx]

**S4 Table. Stunting prevalence (HAZ<-2, HAZ<-3) in children aged 6-59 months^1^**

| **Camp** | **Time point** | **Height-for-age z-score <-2 and ≥-3** | **Height-for-age z-score <-3** |
| --- | --- | --- | --- |
| Dagahaley | Baseline (n=520) | 14.2 (11.4, 17.6) | 6.5 (4.31, 9.81) |
|  | End-line (n=537) | 15.3 (11.5, 20.5) | 5.4 (3.14, 9.14) |
| Hagadera | Baseline (n=600) | 20.8 (17.8, 24.2) | 11.0 (7.92, 15.1) |
|  | End-line (n=574) | 15.5 (12.8, 19.1) | 6.3 (4.73, 8.27) |
| Ifo | Baseline (n=516) | 18.2 (13.9, 23.6) | 6.2 (4.06, 9.37) |
|  | End-line (n=504) | 15.1 (11.5, 19.5) | 8.1 (5.89, 11.1) |
| Kakuma | Baseline (n=559) | 13.8 (10.5, 17.9) | 6.7 (4.70, 10.2) |
|  | End-line (n=604) | 20.0 (16.4, 24.3) | 5.5 (3.70, 7.99) |
| Ali Addeh | Baseline (n=305) | 23.9 (19.5, 29.1) | 18.4 (14.4, 23.1) |
|  | End-line (n=512) | 24.8 (21.0, 29.0) | 16.6 (12.9, 21.1) |

^1^ The data are prevalence % (95% Confidence Interval). Cluster numbers were not available for the baseline survey in Ali Addeh so confidence intervals were calculated without allowing for clustering.
