# Supplementary material for: Assessment of the effectiveness of a small quantity lipid-based nutrient supplement on reducing anaemia and stunting in refugee populations in the Horn of Africa: Secondary data analysis
Source: PLoS One. 2017 Jun 7;12(6):e0177556. doi: 10.1371/journal.pone.0177556 (PMC5462343; doi:10.1371/journal.pone.0177556)
Supplement: S1 Questionnaire — (DOC) [file pone.0177556.s005.doc]

**5. Dadaab Nutrition Survey, September 2011, Questionnaire for Children Aged 6-59 months**

| **Date (dd/mm):**  |______|_____|/|_____|_____|/2011 | | | | | | | | | | **Cluster Number:** |_____|_____| | | | | | | | | **Team Number:** |_____| | | | | | | |
| --- | --- | --- | --- | --- | --- | --- | --- | --- | --- | --- | --- | --- | --- | --- | --- | --- | --- | --- | --- | --- | --- | --- | --- | --- |
| **Block Code / Number:**  |_____|_____|_____| | | | | | | | | | | **Camp/Survey Number:**  1= HAG, 2 = IFO, 3= DAG, 4 = DAG-OS |_____| | | | | | | | | **Inclusion date:________________________________________**  **Exclusion date:________________________________________** | | | | | | |
|  |  | | **C1** | **C2** | | **C3** | | **C4** | | | **C5** | | **C6** | | **C7** | | **C8** | | | **C9** | | **C10** | | |
| **Child**  **No.** | **HH**  **No.** | | **Consent**  1=yes  2=no  3=absent | **Sex**  (m/f) | | **Birthdate**  (dd/mm/yyyy) | | **Age** (months) | | | **Weight**  (kg)  ±100g | | **Height** (cm)  ±0.1cm | | **Oedema**  (y/n) | | **MUAC**  (cm) | | | **Weight taken with clothes**  (y/n) | | **IS CHILD ENROLED IN NUTRITION PROGRAMME?**  1 = OTP  2 = SFP  3 = Referred  4 = Not needed | | |
| 1 |  | |  |  | |  | |  | | |  | |  | |  | |  | | |  | |  | | |
| 2 |  | |  |  | |  | |  | | |  | |  | |  | |  | | |  | |  | | |
| 3 |  | |  |  | |  | |  | | |  | |  | |  | |  | | |  | |  | | |
| 4 |  | |  |  | |  | |  | | |  | |  | |  | |  | | |  | |  | | |
| 5 |  | |  |  | |  | |  | | |  | |  | |  | |  | | |  | |  | | |
| 6 |  | |  |  | |  | |  | | |  | |  | |  | |  | | |  | |  | | |
| 7 |  | |  |  | |  | |  | | |  | |  | |  | |  | | |  | |  | | |
| 8 |  | |  |  | |  | |  | | |  | |  | |  | |  | | |  | |  | | |
| 9 |  | |  |  | |  | |  | | |  | |  | |  | |  | | |  | |  | | |
| 10 |  | |  |  | |  | |  | | |  | |  | |  | |  | | |  | |  | | |
| 11 |  | |  |  | |  | |  | | |  | |  | |  | |  | | |  | |  | | |
| 12 |  | |  |  | |  | |  | | |  | |  | |  | |  | | |  | |  | | |
| 13 |  | |  |  | |  | |  | | |  | |  | |  | |  | | |  | |  | | |
| 14 |  | |  |  | |  | |  | | |  | |  | |  | |  | | |  | |  | | |
| 15 |  | |  |  | |  | |  | | |  | |  | |  | |  | | |  | |  | | |
| 16 |  | |  |  | |  | |  | | |  | |  | |  | |  | | |  | |  | | |
| 17 |  | |  |  | |  | |  | | |  | |  | |  | |  | | |  | |  | | |
| 18 |  | |  |  | |  | |  | | |  | |  | |  | |  | | |  | |  | | |
| 19 |  | |  |  | |  | |  | | |  | |  | |  | |  | | |  | |  | | |
| **IF NO VALID AGE DOCUMENTATION IS AVAILABLE:** DO NOT FILL IN C3 AND ESTIMATE AGE USING LOVAL EVENTS CALENDAR.  **C7 and C8: REFER TO HEALTH POST FOR MALNUTRITION IF NOT ALREADY ENROLED IN SFP /OTP**: REFER IF OEDEMA OR IF MUAC<12.5 cm | | | | | | | | | | | | | | | | | | | | | | | | |
|  |  | **C11** | | | **C12** | **C13** | **C14** | | **C15** | | | **C16** | | **C17** | | **C18** | | | **C19** | | **C20** | | **C21** | **C22** |
| **Child**  **No.** | **HH**  **No.** | **When did [name] arrive in the camps?**  IF ANSWER IS 10 GO TO C13  USE CODES BELOW | | | **Region of origin**  USE CODES BELOW | **Ethnic group**  1=Somali  2=Somali Bantu  98=Other | **Measles Vaccination**  1=yes with card  2=yes recall  3=No with card  4=No recall or don’t know | | **PENTA1 or**  **PENTA2 or**  **PENTA3 with card only**  1=1 dose  2=2 doses  3=3 doses  4=No PENTA or no card | | | **Vit. A in past 6 months**  (SHOW CAPSULE)  1=Yes card  2=Yes recall  3=No or don’t know | | **Dewormed in past 6 months** (SHOW PILL)  1=Yes card  2=Yes recall  3=No or don’t know | | **Diarrhoea in last 2 weeks**  1 = yes  2 = no  GO TO C20  99 = don’t know  GO TO C20 | | | **When [name] had diarrhoea did you feed [name]:**  1=less  2=the same  3=more  4=no food | | **Hb**  (g/dL) | | **Child referred for severe anaemia**  1=yes  2=no | **IS THIS CHILD AGED 6-23 MONTHS?**  1=yes  2=no  STOP NOW |
| 1 |  |  | | |  |  |  | |  | | |  | |  | |  | | |  | |  | |  |  |
| 2 |  |  | | |  |  |  | |  | | |  | |  | |  | | |  | |  | |  |  |
| 3 |  |  | | |  |  |  | |  | | |  | |  | |  | | |  | |  | |  |  |
| 4 |  |  | | |  |  |  | |  | | |  | |  | |  | | |  | |  | |  |  |
| 5 |  |  | | |  |  |  | |  | | |  | |  | |  | | |  | |  | |  |  |
| 6 |  |  | | |  |  |  | |  | | |  | |  | |  | | |  | |  | |  |  |
| 7 |  |  | | |  |  |  | |  | | |  | |  | |  | | |  | |  | |  |  |
| 8 |  |  | | |  |  |  | |  | | |  | |  | |  | | |  | |  | |  |  |
| 9 |  |  | | |  |  |  | |  | | |  | |  | |  | | |  | |  | |  |  |
| 10 |  |  | | |  |  |  | |  | | |  | |  | |  | | |  | |  | |  |  |
| 11 |  |  | | |  |  |  | |  | | |  | |  | |  | | |  | |  | |  |  |
| 12 |  |  | | |  |  |  | |  | | |  | |  | |  | | |  | |  | |  |  |
| 13 |  |  | | |  |  |  | |  | | |  | |  | |  | | |  | |  | |  |  |
| 14 |  |  | | |  |  |  | |  | | |  | |  | |  | | |  | |  | |  |  |
| 15 |  |  | | |  |  |  | |  | | |  | |  | |  | | |  | |  | |  |  |
| 16 |  |  | | |  |  |  | |  | | |  | |  | |  | | |  | |  | |  |  |
| 17 |  |  | | |  |  |  | |  | | |  | |  | |  | | |  | |  | |  |  |
| 18 |  |  | | |  |  |  | |  | | |  | |  | |  | | |  | |  | |  |  |
| 19 |  |  | | |  |  |  | |  | | |  | |  | |  | | |  | |  | |  |  |
| **CODES:**  **C11**: 1 = September, 2 = August, 3 = July, 4 = June, 5 = May, 6 = April, 7 = March, 8 = February, 9 = January, 10 = Before January or born in camp.  **C12**: 1 = Lower Juba, 2 = Middle Juba, 3 = Gedo, 4 = Bay, 5 = Bakool, 6 = Lower Shabelle, 7 = Middle Shabelle, 8 = Hiraan, 9 = Mogadishu / Banadir, 98= Other  **REFERAL TO HEALTH POST FOR SEVERE ANAEMIA - C20:** REFER IF Hb < 7.0 g/dL | | | | | | | | | | | | | | | | | | | | | | | | |

|  |  | **C23** | **C24** | **C25** | **C26** | **C27** | | **C28** | | **C29** | **C30** | | **C31** | | **C32** | **C33** | **C34** | **C35** | **C36** | **C37** | **C38** |
| --- | --- | --- | --- | --- | --- | --- | --- | --- | --- | --- | --- | --- | --- | --- | --- | --- | --- | --- | --- | --- | --- |
| **Child**  **No.** | **HH**  **No.** | **Did you ever breastfeed [name]?**  1 = yes  2 = no  GO TO C26  99 = don’t know  GO TO C26 | **How long after birth did you first put (name) to the breast?**  1= less than 1 hr  2 = >1hr - <24 hrs  3 = ≥24 hrs  99 = don’t know | **Was [name] breastfed yesterday during the day or at night?**  1 = yes  2 = no  99 = don’t know | Now I will ask you about what [name] drank or ate during the day or at night. Yesterday during the day or at night, did [name] receive (INSERT ITEM HERE)?  1= yes, 2 = no, 99 = don’t know | | | | | | | | | | | | | | | | **Did [name] drink anything from a bottle with a nipple yesterday during the day or night?**  1 = yes  2 = no  99 = don’t know |
|  |  |  |  |  | Plain water | Sugar water | Fresh fruit juice | | Sweetened flavoured juices (Zeitun, Altuza, Mushakil, vimto, soda, afya) | | Tea or coffee white or black | Infant formula: for example Mamex, Sahar, Nan, S26 | | Fresh animal milk or any tinned or powdered milk. | | Porridge made from CSB | Porridge not made from CSB | Medicines: for example ORS, gripe water | Nutributter (SHOW SACHET) | Foods other than liquids (semi-solid and solid foods,) |  |
| 1 |  |  |  |  |  |  |  | |  | |  |  | |  | |  |  |  |  |  |  |
| 2 |  |  |  |  |  |  |  | |  | |  |  | |  | |  |  |  |  |  |  |
| 3 |  |  |  |  |  |  |  | |  | |  |  | |  | |  |  |  |  |  |  |
| 4 |  |  |  |  |  |  |  | |  | |  |  | |  | |  |  |  |  |  |  |
| 5 |  |  |  |  |  |  |  | |  | |  |  | |  | |  |  |  |  |  |  |
| 6 |  |  |  |  |  |  |  | |  | |  |  | |  | |  |  |  |  |  |  |
| 7 |  |  |  |  |  |  |  | |  | |  |  | |  | |  |  |  |  |  |  |
| 8 |  |  |  |  |  |  |  | |  | |  |  | |  | |  |  |  |  |  |  |
| 9 |  |  |  |  |  |  |  | |  | |  |  | |  | |  |  |  |  |  |  |
| 10 |  |  |  |  |  |  |  | |  | |  |  | |  | |  |  |  |  |  |  |
| 11 |  |  |  |  |  |  |  | |  | |  |  | |  | |  |  |  |  |  |  |
| 12 |  |  |  |  |  |  |  | |  | |  |  | |  | |  |  |  |  |  |  |
| 13 |  |  |  |  |  |  |  | |  | |  |  | |  | |  |  |  |  |  |  |
| 14 |  |  |  |  |  |  |  | |  | |  |  | |  | |  |  |  |  |  |  |
| 15 |  |  |  |  |  |  |  | |  | |  |  | |  | |  |  |  |  |  |  |
| 16 |  |  |  |  |  |  |  | |  | |  |  | |  | |  |  |  |  |  |  |
| 17 |  |  |  |  |  |  |  | |  | |  |  | |  | |  |  |  |  |  |  |
| 18 |  |  |  |  |  |  |  | |  | |  |  | |  | |  |  |  |  |  |  |
| 19 |  |  |  |  |  |  |  | |  | |  |  | |  | |  |  |  |  |  |  |
| **C37**: For example: pasta, rice, anjera, ugali, potatoes, maize, beans, mango, banana, other fruits and vegetables, meat. | | | | | | | | | | | | | | | | | | | | | |

|  |  | **C39** | **C40** | **C41** | **C42** | **C43** |  |
| --- | --- | --- | --- | --- | --- | --- | --- |
| **Child**  **No.** | **HH**  **No.** | **Is [name] currently receiving Nutributter (*SHOW SACHET*)?**  1=yes  2=no  GO TO C42  99=don’t know  GO TO C42 | **How many sachets did you collect the last time you went to collect Nutributter?**  (number of sachets) | **Is Nutributter eaten by others in the family?**  1=yes  2=no | **IS THIS CHILD AGED 6-12 MONTHS?**  1=yes  2=no  STOP NOW | **Do you currently have a fresh food voucher?**  1=yes  2=no  99=don’t know (ADVISE) |
| 1 |  |  |  |  |  |  |
| 2 |  |  |  |  |  |  |
| 3 |  |  |  |  |  |  |
| 4 |  |  |  |  |  |  |
| 5 |  |  |  |  |  |  |
| 6 |  |  |  |  |  |  |
| 7 |  |  |  |  |  |  |
| 8 |  |  |  |  |  |  |
| 9 |  |  |  |  |  |  |
| 10 |  |  |  |  |  |  |
| 11 |  |  |  |  |  |  |
| 12 |  |  |  |  |  |  |
| 13 |  |  |  |  |  |  |
| 14 |  |  |  |  |  |  |
| 15 |  |  |  |  |  |  |
| 16 |  |  |  |  |  |  |
| 17 |  |  |  |  |  |  |
| 18 |  |  |  |  |  |  |
| 19 |  |  |  |  |  |  |

Team leader signature:____________________________
